# Characterisation of breast fine-needle aspiration biopsies by centrosome aberrations and genomic instability

**DOI:** 10.1038/sj.bjc.6602246

**Published:** 2004-11-23

**Authors:** U Kronenwett, S Huwendiek, J Castro, T Ried, G Auer

**Affiliations:** 1 Department of Oncology and Pathology, Division of Cellular and Molecular Analysis, Cancer Center Karolinska (CCK), R8:04, Karolinska Institute and Hospital, 17176 Stockholm, Sweden; 2 Genetics Branch, Center for Cancer Research, National Cancer Institute, National Institutes of Health, Bethesda, MD 20892-0913, USA

**Keywords:** breast tumours, DCIS, FNAB, image cytometry, SSI, centrosome aberrations

## Abstract

Recent studies have suggested that aneuploidy in malignant tumours could be a consequence of centrosome aberrations. Using immunofluorescence analysis with an antibody against *γ*-tubulin and DNA image cytometry, we measured centrosome aberrations and DNA ploidy patterns in fine-needle aspiration biopsies (FNABs) of 58 breast lesions. Benign lesions did not show any centrosome aberrations. DNA diploid carcinomas showed a mean percentage of cells with centrosomal defects of 2.1%. The aneuploid invasive carcinomas could be divided into two subgroups by their significantly (*P=*0.0003) different percentage of cells with centrosome aberrations (2.0 and 10.3%, respectively) and their significantly (*P=*0.0003) different percentage of cells with nonmodal DNA content values determined by the Stemline Scatter Index (SSI), a measure of genomic instability. The percentage of cells with centrosome aberrations demonstrated a positive, linear correlation with the corresponding SSI (*r*=0.82, *P*<0.0001) and loss of tissue differentiation (*r*=0.78, *P*<0.0001). Our results indicate the percentage of cells with centrosome aberrations as being sufficient to divide the investigated tumours into three significantly different groups: benign lesions with no centrosomal aberrations, and two malignant tumour types with mean values of 2.1 and 9.6% of centrosomal defects, respectively. Together, these results demonstrate that centrosome aberrations correlate with genomic instability and loss of tissue differentiation. Furthermore, this study shows the feasibility of centrosomal analysis in FNAB of the breast and suggests centrosomal aberrations as possessing diagnostic and prognostic value.

Fine-needle aspiration biopsy (FNAB) is a well established, low cost, rapid and minimally invasive technique for diagnosing suspicious breast lesions that can reduce the need of surgical biopsies by preoperative diagnosis (Franzen and Zajicek, 1968; Arisio *et al*, 1998). The accuracy through which lesions can be targeted has been improved by sonography and mammography guided aspiration techniques. However, diagnosis in cytological specimens is in general more difficult than in histological ones, because information about epithelial cell growth pattern and connection with the surrounding tissue are lost. Consequently, diagnosing cytological samples, in particular from early and highly differentiated carcinomas, remains challenging not only due to the absence of histomorphological structures but also because of the frequently minute cytomorphological alterations. Hence, the requirement of additional and objective information is of utmost priority. Centrosome aberrations seem to be characteristic of malignant tumours in general (Pihan *et al*, 1998). Normally, the centrosome as the major microtubule organisation centre in mammalian cells regulates the number, stability, polarity and arrangement of microtubules in interphase cells (Rose *et al*, 1993; Kellogg *et al*, 1994). In this context, the centrosome and microtubules play an important role in maintaining overall cell polarity and provide an architectural framework for several cell functions. Once in each cell cycle the centrosome duplicates itself (Stearns, 2001). During G2–M phase transition, replicated centrosomes separate to guarantee correct chromosome segregation by formation of a bipolar mitotic spindle (Kellogg *et al*, 1994; Meraldi *et al*, 1999). Under normal conditions, the centrosome duplication cycle proceeds in exact coordination with the DNA replication (Sluder and Hinchcliffe, 2000; Hinchcliffe and Sluder, 2001). Failure of coordination of centrosome duplication and DNA replication may lead to centrosome aberrations, possibly followed by segregation errors of chromosomes at cell division, and thus to aneuploidy and genomic instability (Brinkley and Goepfert, 1998; Orr-Weaver and Weinberg, 1998; Ghadimi *et al*, 2000).

In this study we focused on primary breast carcinomas, which are classically divided into two major groups, that is, DNA diploid and DNA aneuploid tumours by means of DNA content-based histogram analysis. A number of prospective and retrospective studies revealed DNA aneuploid breast carcinomas as following a more malignant course than DNA diploid breast carcinomas do (Auer *et al*, 1980; 1984; Fallenius *et al*, 1988b). Recently, we have shown that aneuploid breast cancers can be subdivided into a genomically stable and unstable group by their proportion of cells with nonmodal DNA content values in the respective cytometrical DNA histograms, where the stable group proved to have a more favourable prognosis (Kronenwett *et al*, 2004). Here we show the feasibility of centrosome analysis in fine-needle aspirates of breast lesions. We explore the relationship between centrosome defects (aberrant centrosome number and size), DNA ploidy and genomic instability.

## MATERIALS AND METHODS

### Patient samples

In total, 51 consecutive FNABs of breast lesions, including 33 invasive carcinomas and 18 benign lesions, were collected at Cell and Molecular Analysis, Karolinska Hospital, during 2000–2001. Furthermore, seven FNABs of ductal carcinomas *in situ* (DCIS) were included in our analysis to characterise preinvasive lesions. The invasive carcinomas comprised 31 ductal carcinomas (DC), one lobular carcinoma (LC) and one medullar carcinoma (MC). The benign lesions consisted of 10 mastopathias and eight fibroadenomas. None of the patients received any type of therapeutic treatment before FNAB. The aspirates were subjected to cytopathological assessment, centrosomal analysis and image cytometric DNA content measurement. All cytologically diagnosed malignancies and all suspicious lesions were operated and examined histopathologically (Table 1).

### FNAB

FNAB was performed using a 23-gauge needle attached to a 10 ml syringe and inserted into a syringe holder as described earlier (Franzen and Zajicek, 1968; Auer *et al*, 1980). All aspirations were performed by the same experienced pathologist (GA). Cells from nonpalpable lesions were aspirated under guidance of ultrasound or mammography (Fornage *et al*, 1992). The aspirates were smeared on microscope slides for cytopathological evaluation after May–Gruenwald–Giemsa staining.

### DNA image cytometry

Image cytometry was performed on Feulgen stained FNABs to measure the nuclear DNA content. The staining, the internal standardisation and the tumour cell selection were based on previously described methods (Auer *et al*, 1980). All DNA values were calculated in relation to a corresponding staining control, which obtained the value 2c, denoting the diploid DNA content. On average, we measured 350 morphologically selected cells per aspirate. The cell population of each specimen was characterised according to two principles, based upon nuclear DNA content measurements. First, we divided the tumours into the already known groups: type I, with a diploid stemline (1.8c–2.2c), <5% of the cells in S phase and no cells exceeding 5c; type II, with a tetraploid stemline (3.8c–4.2c) and <5% of the cells exceeding 5c; type III or diploid proliferative, with a diploid stemline, but ⩾5% of the cells being in S phase; and type IV or aneuploid, with one peak or more outside the diploid or tetraploid region (Auer *et al*, 1980; 1989). Furthermore, we applied the Stemline Scatter Index (SSI) to the 58 breast lesions, a measure of the percentage of tumour cells with nonmodal DNA content values, or of the degree of scattering of DNA histograms (Kronenwett *et al*, 2004). The SSI is the sum of the percentage of cells with DNA content values in the S-phase region (S phase), plus the percentage of cells with DNA content values exceeding twice the modal value plus 1c (G2 exceeding rate, or G2 Exc), plus the coefficient of variation (CV) of the respective tumour stemline (SSI=S phase+G2 Exc+Tum CV). We used the cut point value of SSI=8.8% (Kronenwett *et al*, 2004) to differentiate between all lesions showing significantly scattered DNA histograms (SSI>8.8%) and those with insignificantly scattered ones (SSI⩽8.8%). Breast lesions with an SSI ⩽8.8% were termed genomically stable (gs), and those with an SSI>8.8% were termed genomically unstable (gu).

### Centrosome staining

The FNABs were smeared on a glass slide and shortly air-dried. Then the aspirated cells were fixed in methanol at −20°C for 10 min and in acetone at −20°C for 6 min. Blocking solution (1% normal goat serum (NGS) and 0.1% Tween 20 in PBS) was applied to the cells for 30 min at 37°C. Cells were incubated with an anti-*γ*-tubulin monoclonal antibody (Sigma-Aldrich, St Louis; diluted 1 : 1000 in PBS, containing 2% NGS) for 1 h. Antibody–antigen complexes were detected by Cy3-conjugated anti-mouse IgG (Jackson Immuno Research, West Grove; 1 : 200, 2% NGS/PBS for 1 h at room temperature). After each incubation, cells were extensively rinsed with PBS. Then DNA was visualised by staining with DAPI (4′,6-diamino 2-phenylindole, Sigma-Aldrich) for 5 min at room temperature. After washing in PBS for 5 min, the stained slides were mounted with mounting medium (Vectashield, Vector Laboratories, Peterborough, UK) and covered with a cover slide. The immunofluorescence staining was evaluated using a fluorescence microscope (Leica DM RXA), and the centrosome images were obtained with the aid of Leica Q-FISH software. The percentage of cells with centrosome aberrations was determined by dividing the number of cells showing more than two centrosomes (=*γ*-tubulin-stained spots), or centrosomes with apparently aberrant morphology (a diameter of greater than twice the diameter of centrosomes of nontumour control cells) by the number of investigated cells. At least 500 cells per aspirate were examined. All slides were coded and evaluated by two investigators independently.

### Statistical analysis

The significance of correlation of centrosome aberrations with SSI and centrosome aberrations with Elston grade were assessed according to Spearman. For group discrimination significance analysis, the *U*-Test (Mann–Whitney) was applied. All statistics were calculated with the aid of SPSS.

## RESULTS

In this study 31 DC, one LC, one MC, seven DCIS, 10 mastopathias and eight fibroadenomas were characterised by their centrosomal status and their cytometrical DNA content histogram. All samples were obtained by FNAB.

### DNA image analysis

In total, 65% (20 out of 31) of the DC, the one (one out of one) MC and all (seven out of seven) DCIS were aneuploid (A), whereas 35% (11 out of 31) of the DC, the one (one out of one) LC and all (18 out of 18) benign lesions were diploid (D). Despite the fact that all DCIS in this study were A, a previous study from our group showed 63% of DCIS as being A and 37% as being D (Pallis *et al*, 1992).

**Figure 1:**
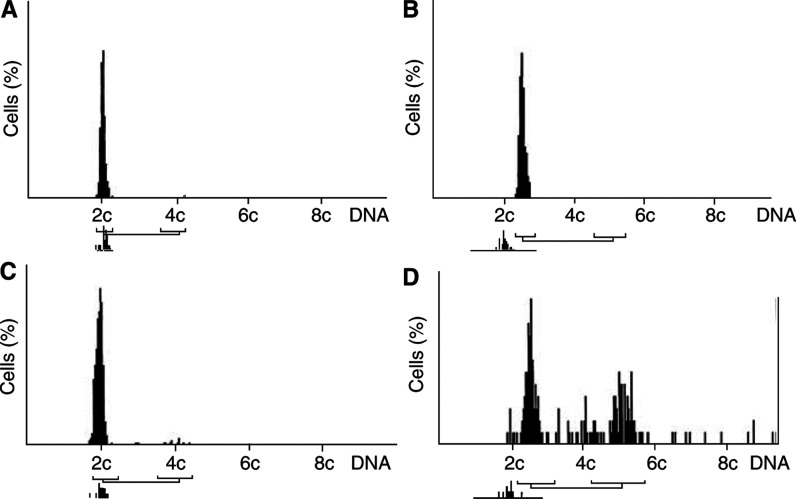
Diploid and aneuploid DNA histograms of fine-needle aspiration biopsies of the breast. Nuclear DNA content of the cells on the horizontal axis is normalised to the nuclear DNA content of leukocytes (2c denotes diploid DNA content). (**A**) DNA profile of a fibroadenoma, (**B**) profile of a genomically stable (gs) aneuploid carcinoma, (**C**) DNA histogram of a gs diploid carcinoma, and (**D**) profile of a genomically unstable aneuploid carcinoma.

By using the cut point value of SSI=8.8%, we determined seven Ags tumours (gs: SSI ⩽8.8%, Figure 1B) and 14 Agu specimens (gu: SSI>8.8%, Figure 1D) among the invasive A carcinomas. The seven DCIS were found to be Agu as well. The invasive D carcinomas (*n*=12, Figure 1C) and benign D lesions (*n*=18, Figure 1A) were all gs.

### Centrosomal status

**Figure 2:**
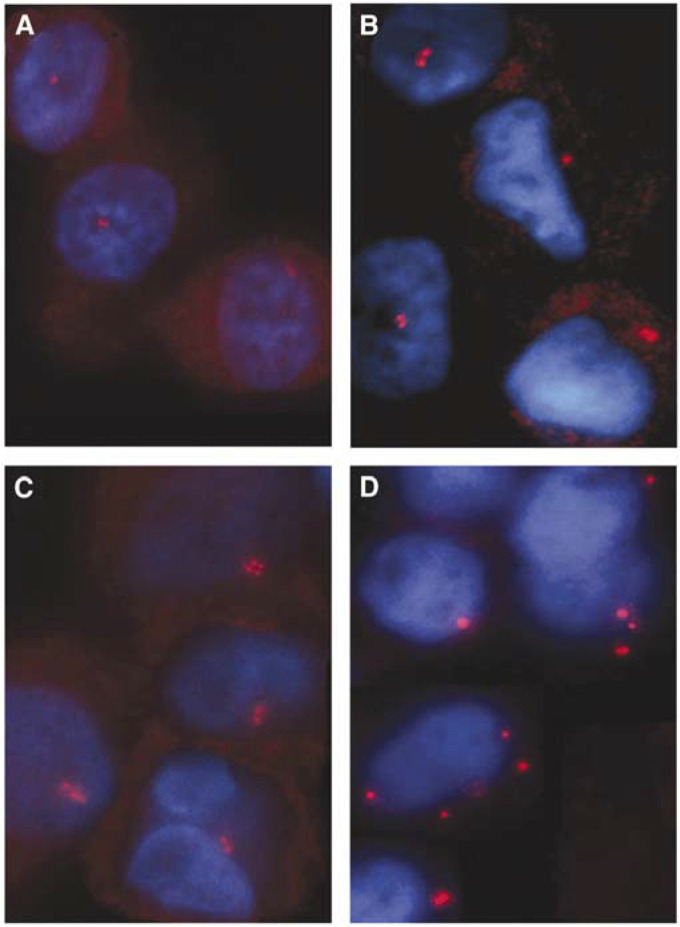
Centrosome staining of fine-needle aspiration biopsies of genomically stable (gs) breast lesions. Centrosomes are immunostained with a monoclonal antibody against *γ*-tubulin. The fibroadenoma (**A**) shows no centrosome aberrations, that is, two centrosomes (red) per nucleus (blue). gs diploid (**B**, **C**) and gs aneuploid (**D**) carcinomas show a low range of supernumerary centrosomes and occasionally centrosomes with larger size (**B**).

**Figure 3:**
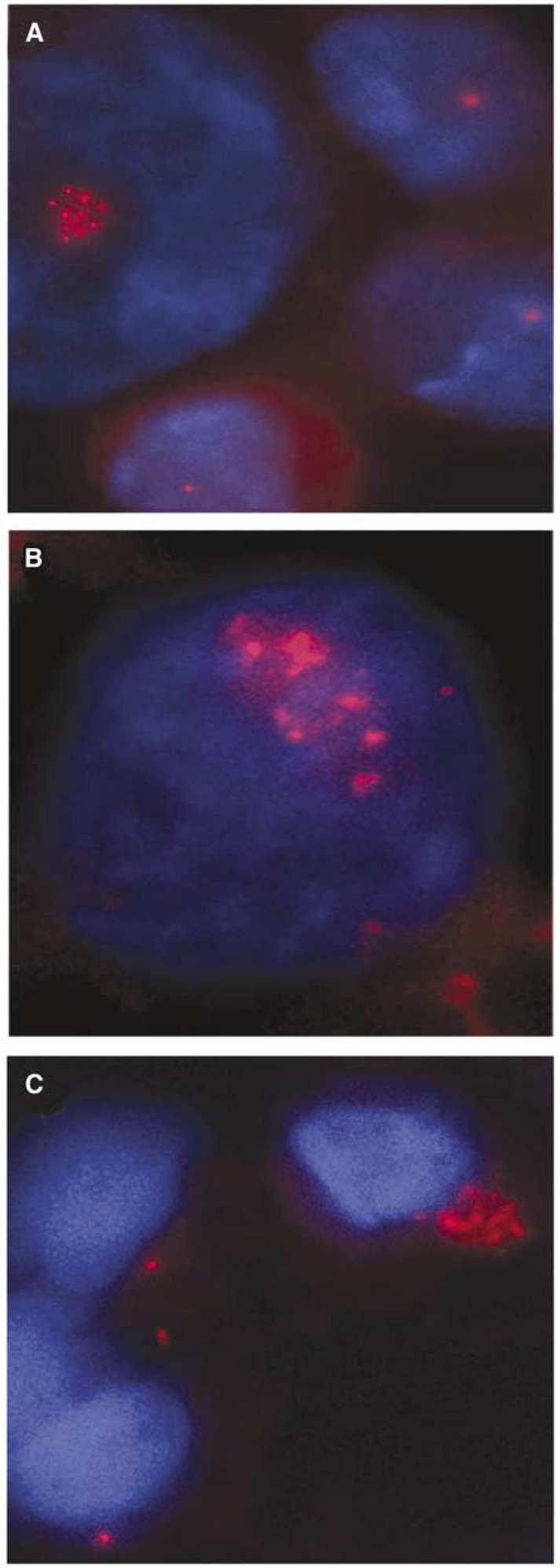
Centrosome staining of fine-needle aspiration biopsies of genomically unstable (gu) aneuploid breast carcinomas. Centrosomes are immunostained with a monoclonal antibody against *γ*-tubulin. gu aneuploid carcinomas (**A–C**) demonstrate a high range of supernumerary centrosomes (red spots). Centrosomes are more often of larger size (**B**, **C**) than in genomically stable tumours.

The tumour aspirates were immunostained with an antibody against *γ*-tubulin, a well-characterised marker of the centrosome (Stearns *et al*, 1991; Joshi *et al*, 1992; Schiebel, 2000). Centrosome aberrations were investigated by the determination of the percentage of cells with more than two centrosomes (*γ*-tubulin-stained spots) and centrosomes with apparently aberrant morphology: a diameter of greater than twice the diameter of centrosomes of normal breast cells and fibroblasts in imprints. The investigated aspirates differed distinctly concerning the extent of centrosome defects. None of the benign lesions (10 mastopathias and eight fibroadenomas) had any centrosome aberrations (0%, Figure 2A). The Ags carcinomas (*n*=7) showed a mean percentage of cells with centrosome aberrations of 2.0% (s.d.=0.26, Figure 2D), similar to that in Dgs carcinomas (*n*=12) with a mean percentage of 2.1% (s.d.=0.26, Figure 2B and C). The invasive Agu breast carcinomas (*n*=14) had the highest percentage of cells with centrosomal aberrations with a mean value of 10.3% (s.d.=1.66, Figure 3A–C). The corresponding value in the Agu DCIS specimens (*n*=7) was 8.2% (s.d.=1.35). Most frequently observed abnormalities were cells with supernumerary centrosomes (range: 3–11).

**Figure 4:**
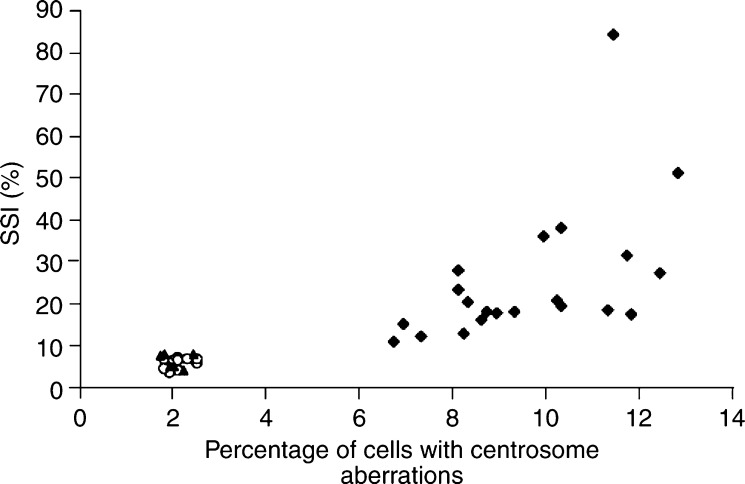
Three significantly different groups of breast lesions regarding the centrosomal status: (1) benign (▪) no centrosomal aberrations; (2) malignant with a low extent of cells with centrosomal aberrations: genomically stable (gs) diploid (○) and gs aneuploid (▴); (3) malignant with a higher extent of cells with centrosomal aberrations: invasive genomically unstable (gu) aneuploid and gu aneuploid *in situ* (♦). There is a positive linear correlation between SSI (Stemline Scatter Index) and the percentage of cells with centrosome aberrations (*r*=0.82, *P*<0.0001).

The investigated specimens could be divided into three significantly different groups, regarding their centrosomal status: (1) benign lesions with no measurable centrosomal aberrations; (2) malignant tumours with a low extent of cells with centrosomal aberrations, including Dgs and Ags carcinomas with a mean value of centrosome aberrations of 2.1% (s.d.=0.26); and (3) malignant tumours with a higher extent of cells with centrosomal defects, including invasive Agu and Agu DCIS with a mean value of 9.6% (s.d.=1.82) (Figure 4, Table 2). Furthermore, the invasive A carcinomas could be divided into two subgroups (Agu and Ags) by their significantly different percentage of cells with centrosome aberrations and their significantly different fraction of cells with nonmodal DNA content values measured by the SSI (Table 2). The significance of differences between the aforementioned groups is indicated in Table 3. Our results also permit division of the FNAB specimens into the same three groups from above, using the SSI as separation criterion (Tables 2 and 3). However, to separate benign from malignant breast tumours, we need to investigate a larger number of benign lesions, in order to determine a cut point of SSI between these two groups and to be able to validate the cut point.

We found a significant, linear correlation between SSI and percentage of cells with centrosome aberrations (*r*=0.82, *P*<0.0001). Also, the percentage of cells with centrosomal anomalies did correlate with increased Elston grade in the invasive carcinomas (*r*=0.78, *P*<0.0001), suggesting a relationship between centrosomal aberrations and cytological events, leading to loss of tissue differentiation.

## DISCUSSION

In this study, we characterised 58 FNABs of breast lesions by their centrosomal status and DNA content-based histogram. We found that centrosome aberrations correlate with genomic instability, as indicated by a high percentage of cells with nonmodal DNA content values, measured by the SSI, and loss of tissue differentiation. Already preinvasive lesions showed centrosomal aberrations, suggesting centrosome abnormalities as being an early step in breast carcinogenesis. The investigated breast lesions could be divided into benign lesions (no centrosomal aberrations) and two subgroups of malignant tumours, by their significantly different extent of cells with centrosomal aberrations.

Image cytometry measurements are carried out to identify aneuploidy, which is the most frequent manifestation of genomic instability in human malignancies (Mertens *et al*, 1997; Sen, 2000). Type IV histograms, those of aneuploid tumours (Auer *et al*, 1980), are suggested to indicate populations of interphase nuclei with decreased genomic stability (Auer *et al*, 1989). Although DNA ploidy measurement does not reveal information about the distribution of genomic imbalances, several studies using this method have supported the conclusion that DNA aneuploidy is closely associated with poor prognosis in various cancers (Ross, 1996; Magennis, 1997). In breast cancer the prognostic significance of DNA content has been shown in several studies (Auer *et al*, 1984; Fallenius *et al*, 1988a; 1988b; Siitonen *et al*, 1993). Using Cox multivariate analysis, nuclear DNA content was found to provide significant prognostic information, independent of any other clinical and histomorphological variables (Fallenius *et al*, 1988a).

In the present work, we classified breast tumours by their DNA content histograms. Applying the SSI value (Kronenwett *et al*, 2004), we were able to further subdivide aneuploid breast lesions into gs and gu types. We found that scattering of DNA profiles as a measure of genomic instability correlates highly significantly with centrosome aberrations. While centrosome abnormalities have been reported earlier in different human malignancies (Pihan *et al*, 1998; Ghadimi *et al*, 2000; Skyldberg *et al*, 2001; Roshani *et al*, 2002), including breast cancer (Lingle *et al*, 1998; Lingle and Salisbury, 1999), a difference in the extent of centrosome aberrations in cytometrically determined subtypes of breast carcinomas has, to our knowledge, not been reported by other authors.

None of the benign breast lesions showed any centrosomal defects. In contrast, all carcinomas exhibited centrosomal aberrations. Interestingly, invasive gs diploid and gs aneuploid carcinomas demonstrated a comparable percentage of cells with centrosomal abnormalities (mean value 2.1%), which differed distinctly from the approximately five times higher value, observed in the invasive gu aneuploid tumours (mean value 10.3%). These results confirm our data received from a former study, where we investigated three gs aneuploid and diploid breast carcinomas each, and four gu aneuploid ones (Kronenwett *et al*, 2004).

In accordance with findings from other authors (Lingle *et al*, 2002; Pihan *et al*, 2003), we detected centrosome aberrations already in noninvasive breast lesions (DCIS), suggesting centrosome aberrations as being an early event in the complex process of breast carcinogenesis. The range of centrosome aberrations in the DCIS was very similar (even though slightly lower) to that of their invasive counterparts and consistent with their ploidy pattern. Using fluorescence *in situ* hybridisation with probes for chromosomes 3, 7 and 17,Lingle *et al* (2002) could distinguish between stable and unstable aneuploid breast carcinomas. The stable specimens possessed only few defects in centrosome number and size, which in turn correlated with chromosomal stability. These findings are in accordance with our results. The gs breast carcinomas characterised by us still seem to have a quite well-regulated cell cycle as shown in *cyclins A* and *E* mRNA expression studies, and they possess only a low percentage (⩽8.8%) of cells with nonmodal DNA content values, indicating a homogeneous tumour cell population. Furthermore, they prove to have a better prognosis than the gu breast carcinomas (Kronenwett *et al*, 2004). Centrosome aberrations in gs aneuploid specimens are in the range of those found in gs diploid ones.

Comparative genomic hybridisation analysis of DNA diploid breast carcinomas revealed few copy number changes that involve mainly the gain or loss of entire chromosomes or chromosomal arms (Ried *et al*, 1995), an observation that may indicate a segregation error as a primary event in carcinogenesis. Such segregation errors could be the consequence of supernumerary centrosomes. An electron microscopy study (Lingle and Salisbury, 1999) revealed that multiple centrosomes in breast cancer cells could lead to multipolar mitosis or via coalescence of two or more centrosomes to bipolar spindles. Thus, the existence of multiple centrosomes might promote genomic instability by increasing the probability of chromosomal segregation errors to occur, whereas through coalescence of excess centrosomes a proportion of bipolar mitosis for tumour growth is maintained. Consequently, centrosome aberrations might play an important role in carcinogenesis and in malignant tumours promote further karyotypic heterogeneity. This is supported by our findings, showing that centrosome aberrations are already present in *in situ* lesions, that is, before invasiveness. Additionally, centrosomal aberrations correlate with genomic instability (as mirrored in the degree of scattering of DNA histograms measured by the SSI) and with loss of tissue differentiation.

All patient samples used for centrosomal and DNA content analysis were obtained with the aid of FNAB. While DNA ploidy measurements are frequently performed on aspirates, to our knowledge this is the first report, which demonstrates the feasibility of centrosomal analysis in FNAB of the breast.

Benign breast diseases, such as mastopathia and fibroadenoma, did not reveal any centrosomal aberrations. Thus, with only one single marker – the percentage of cells with centrosome aberrations – benign lesions were readily discernable from carcinomas/DCIS. Centrosome aberrations revealed in this context more information than did DNA ploidy measurements, because ploidy measurements cannot discriminate benign and malignant diploid lesions. In addition, determining the extent of cells with centrosomal aberrations enabled us to divide the breast cancers into two significantly different subgroups, namely genomically stable and unstable carcinomas.

In a former study, high SSI values were related to unfavourable prognosis (Kronenwett *et al*, 2004). The percentage of cells with centrosome aberrations shows a positive linear correlation with the SSI and, therefore, might be of prognostic value. Further investigations in a larger collective of patients and a sufficient follow-up period are needed to prove the diagnostic and prognostic value of centrosome aberrations in FNABs of the breast.

In summary, our results demonstrate centrosome aberrations as correlating with genomic instability manifested in a high percentage of nonmodal DNA content values, and loss of tissue differentiation. Our results further indicate that centrosome abnormalities might be an early event in the development of breast cancer. The study confirms our previous data (Kronenwett *et al*, 2004), where gs and gu aneuploid breast lesions differed significantly from each other, and gs diploid and gs aneuploid specimens did not. While this has been mainly demonstrated by DNA histogram analysis and *cyclin A* and *E* expression studies before, we show here the two aneuploid subgroups as differing significantly in their centrosomal status as well. Thus, with only one parameter – the percentage of cells with centrosomal aberrations – we could clearly discriminate benign from malignant breast lesions; and in the malignant group, we were able to distinguish two significantly different subgroups. Our results indicate that the characterisation of FNABs of the breast by centrosomal analysis and image cytometry, as a low cost but highly informative method, might support diagnosis and prognosis of breast tumours.

## Figures and Tables

**Table 1:** Patient age and breast cancer characteristics

Age (years)	59 (28–87)
*Histological type*	
Ductal	31 (53.5%)
Lobular	1 (1.7%)
Medullar	1 (1.7%)
DCIS^a^	7 (12.1%)
Mastopathia	10 (17.2%)
Fibroadenoma	8 (13.8%)
	
*Tumour size (carcinomas and DCIS)* b	
T1b	7 (17.5%)
T1c	26 (65.0%)
T2	6 (15.0%)
Not determined	1 (2.5%)
	
*Grade (invasive carcinomas)* c	
Elston Grade 1	6 (18.2%)
Elston Grade 2	17 (51.5%)
Elston Grade 3	10 (30.3%)

a Classification according to Ottesen *et al* (1992).

b Tumour-Node-Metastasis classification (Union International Contre Cancer) 1997.

c Elston–Ellis modification of Scarff–Bloom–Richardson grading system (CW Elston and JO Ellis).

**Table 2:** Centrosome aberrations and stemline scatter index (SSI)

		**Percentage of cells with centrosome aberrations**				**SSI (%)**			
	***n***	**Mean**	**(95% confidence interval)**	**Median**	**s.d.**	**Mean**	**(95% confidence interval)**	**Median**	**s.d.**
All	58	4.14	(3.00–5.29)	2.10	4.35	12.68	(9.02–16.33)	6.50	13.90
(a) Benign lesions	18	0		0	0	4.88	(4.23–5.53)	4.70	1.31
									
*(b) D* a *gs* b *+A* c *gs*	19	2.06	(1.93–2.18)	2.00	0.26	5.90	(5.25–6.55)	6.20	1.37
Dgs	12	2.12	(1.95–2.28)	2.10	0.26	5.65	(4.91–6.39)	6.15	1.17
Ags	7	1.96	(1.71–2.20)	1.90	0.26	6.33	(4.82–7.84)	7.40	1.64
									
*(c) Agu*^d^ *invasive+Agu DCIS*^e^	21	9.58	(8.75–10.41)	9.30	1.82	25.49	(17.93–33.06)	19.40	16.62
Agu invasive	14	10.26	(9.30–11.22)	10.25	1.66	28.59	(17.72–39.46)	20.45	18.83
Agu DCIS	7	8.21	(6.97–9.46)	8.10	1.35	19.30	(10.74–27.86)	17.80	9.25

a Type I carcinomas with a diploid (D) stemline.

b Genomically stable (gs) tumours.

c Type IV carcinomas with an aneuploid (A) stemline.

d Genomically unstable (gu) tumours.

e Ductal Carcinoma *In Situ* (DCIS).

**Table 3:** Mann–Whitney *U*-test of significance of group differences

**Tested groups**	**Centrosome aberrations**	**Stemline Scatter Index (SSI)**
a^a^–b^b^	*P<*0.0001	*P=*0.0308
a–c^c^	*P<*0.0001	*P<*0.0001
b–c	*P<*0.0001	*P<*0.0001
c–A^d^gs^e^	P<0.0001	*P*<0.0001
Agu^f^ invasive – Ags	*P*=0.0003	*P*=0.0003
Agu invasive – D^g^gs	*P*<0.0001	*P*<0.0001

a Benign lesions.

b Genomically stable (gs) type I carcinomas with a diploid (D) stemline and gs type IV carcinomas with an aneuploid (A) stemline.

c Genomically unstable (gu) invasive A carcinomas and Agu carcinomas *in situ.*

d Type IV carcinomas with an aneuploid (A) stemline.

e Genomically stable (gs) tumours.

f Genomically unstable (gu) tumours.

g Type I carcinomas with a diploid (D) stemline.

## References

[bib1] Arisio R, Cuccorese C, Accinelli G, Mano MP, Bordon R, Fessia L (1998) Role of fine-needle aspiration biopsy in breast lesions: analysis of a series of 4.110 cases. Diagn Cytopathol 18: 462–467 9626523 10.1002/(sici)1097-0339(199806)18:6<462::aid-dc16>3.0.co;2-f

[bib2] Auer G, Askensten U, Ahrens O (1989) Cytophotometry. Hum Pathol 20: 518–527 2470665 10.1016/0046-8177(89)90243-8

[bib3] Auer G, Eriksson E, Azavedo E, Caspersson T, Wallgren A (1984) Prognostic significance of nuclear DNA content in mammary adenocarcinomas in humans. Cancer Res 44: 394–396 6690053

[bib4] Auer GU, Caspersson TO, Wallgren AS (1980) DNA content and survival in mammary carcinoma. Anal Quant Cytol 2: 161–165 6252802

[bib5] Brinkley BR, Goepfert TM (1998) Supernumerary centrosomes and cancer: Boveri's hypothesis resurrected. Cell Motil Cytoskeleton 41: 281–288 9858153 10.1002/(SICI)1097-0169(1998)41:4<281::AID-CM1>3.0.CO;2-C

[bib6] Fallenius AG, Auer GU, Carstensen JM (1988a) Prognostic significance of DNA measurements in 409 consecutive breast cancer patients. Cancer 62: 331–341 3383134 10.1002/1097-0142(19880715)62:2<331::aid-cncr2820620218>3.0.co;2-8

[bib7] Fallenius AG, Franzen SA, Auer GU (1988b) Predictive value of nuclear DNA content in breast cancer in relation to clinical and morphologic factors. A retrospective study of 227 consecutive cases. Cancer 62: 521–530 3390793 10.1002/1097-0142(19880801)62:3<521::aid-cncr2820620314>3.0.co;2-f

[bib8] Fornage BD, Coan JD, David CL (1992) Ultrasound-guided needle biopsy of the breast and other interventional procedures. Radiol Clin N Am 30: 167–185 1732925

[bib9] Franzen S, Zajicek J (1968) Aspiration biopsy in diagnosis of palpable lesions of the breast. Critical review of 3479 consecutive biopsies. Acta Radiol Ther Phys Biol 7: 241–262 4179434 10.3109/02841866809133198

[bib10] Ghadimi BM, Sackett DL, Difilippantonio MJ, Schrock E, Neumann T, Jauho A, Auer G, Ried T (2000) Centrosome amplification and instability occurs exclusively in aneuploid, but not in diploid colorectal cancer cell lines, and correlates with numerical chromosomal aberrations. Genes Chromosomes Cancer 27: 183–190 10612807 PMC4721570

[bib11] Hinchcliffe EH, Sluder G (2001) ‘It takes two to tango’: understanding how centrosome duplication is regulated throughout the cell cycle. Genes Dev 15: 1167–1181 11358861 10.1101/gad.894001

[bib12] Joshi HC, Palacios MJ, McNamara L, Cleveland DW (1992) Gamma-tubulin is a centrosomal protein required for cell cycle-dependent microtubule nucleation. Nature 356: 80–83 1538786 10.1038/356080a0

[bib13] Kellogg DR, Moritz M, Alberts BM (1994) The centrosome and cellular organization. Annu Rev Biochem 63: 639–674 7979251 10.1146/annurev.bi.63.070194.003231

[bib14] Kronenwett U, Huwendiek S, Ostring C, Portwood N, Roblick UJ, Pawitan Y, Alaiya A, Sennerstam R, Zetterberg A, Auer G (2004) Improved grading of breast adenocarcinomas based on genomic instability. Cancer Res 64: 904–909 14871819 10.1158/0008-5472.can-03-2451

[bib15] Lingle WL, Barrett SL, Negron VC, D'Assoro AB, Boeneman K, Liu W, Whitehead CM, Reynolds C, Salisbury JL (2002) Centrosome amplification drives chromosomal instability in breast tumor development. Proc Natl Acad Sci USA 99: 1978–1983 11830638 10.1073/pnas.032479999 PMC122305

[bib16] Lingle WL, Lutz WH, Ingle JN, Maihle NJ, Salisbury JL (1998) Centrosome hypertrophy in human breast tumors: implications for genomic stability and cell polarity. Proc Natl Acad Sci USA 95: 2950–2955 9501196 10.1073/pnas.95.6.2950 PMC19675

[bib17] Lingle WL, Salisbury JL (1999) Altered centrosome structure is associated with abnormal mitoses in human breast tumors. Am J Pathol 155: 1941–1951 10595924 10.1016/S0002-9440(10)65513-7 PMC1866918

[bib18] Magennis DP (1997) Nuclear DNA in histological and cytological specimens: measurement and prognostic significance. Br J Biomed Sci 54: 140–148 9231461

[bib19] Meraldi P, Lukas J, Fry AM, Bartek J, Nigg EA (1999) Centrosome duplication in mammalian somatic cells requires E2F and Cdk2-cyclin A. Nat Cell Biol 1: 88–93 10559879 10.1038/10054

[bib20] Mertens F, Johansson B, Hoglund M, Mitelman F (1997) Chromosomal imbalance maps of malignant solid tumors: a cytogenetic survey of 3185 neoplasms. Cancer Res 57: 2765–2780 9205089

[bib21] Orr-Weaver TL, Weinberg RA (1998) A checkpoint on the road to cancer. Nature 392: 223–224 9521314 10.1038/32520

[bib22] Pallis L, Skoog L, Falkmer U, Wilking N, Rutquist LE, Auer G, Cedermark B (1992) The DNA profile of breast cancer *in situ*. Eur J Surg Oncol 18: 108–111 1582502

[bib23] Pihan GA, Purohit A, Wallace J, Knecht H, Woda B, Quesenberry P, Doxsey SJ (1998) Centrosome defects and genetic instability in malignant tumors. Cancer Res 58: 3974–3985 9731511

[bib24] Pihan GA, Wallace J, Zhou Y, Doxsey SJ (2003) Centrosome abnormalities and chromosome instability occur together in pre-invasive carcinomas. Cancer Res 63: 1398–1404 12649205

[bib25] Ried T, Just KE, Holtgreve-Grez H, du Manoir S, Speicher MR, Schrock E, Latham C, Blegen H, Zetterberg A, Cremer T, Auer G (1995) Comparative genomic hybridization of formalin-fixed, paraffin-embedded breast tumors reveals different patterns of chromosomal gains and losses in fibroadenomas and diploid and aneuploid carcinomas. Cancer Res 55: 5415–5423 7585611

[bib26] Rose MD, Biggins S, Satterwhite LL (1993) Unravelling the tangled web at the microtubule-organizing center. Curr Opin Cell Biol 5: 105–115 8448021 10.1016/s0955-0674(05)80015-8

[bib27] Roshani L, Fujioka K, Auer G, Kjellman M, Lagercrantz S, Larsson C (2002) Aberrations of centrosomes in adrenocortical tumors. Int J Oncol 20: 1161–1165 12011993

[bib28] Ross JS (1996) DNA ploidy and cell cycle analysis in cancer diagnosis and prognosis. Oncology (Huntington) 10: 867–882, 887, discussion 887–890 8823802

[bib29] Schiebel E (2000) gamma-tubulin complexes: binding to the centrosome, regulation and microtubule nucleation. Curr Opin Cell Biol 12: 113–118 10679351 10.1016/s0955-0674(99)00064-2

[bib30] Sen S (2000) Aneuploidy and cancer. Curr Opin Oncol 12: 82–88 10687734 10.1097/00001622-200001000-00014

[bib31] Siitonen SM, Kallioniemi OP, Helin HJ, Isola JJ (1993) Prognostic value of cells with more than 5c DNA content in node-negative breast cancer as determined by image cytometry from tissue sections. Hum Pathol 24: 1348–1353 8276382 10.1016/0046-8177(93)90269-m

[bib32] Skyldberg B, Fujioka K, Hellstrom AC, Sylven L, Moberger B, Auer G (2001) Human papillomavirus infection, centrosome aberration, and genetic stability in cervical lesions. Mod Pathol 14: 279–284 11301343 10.1038/modpathol.3880303

[bib33] Sluder G, Hinchcliffe EH (2000) The coordination of centrosome reproduction with nuclear events during the cell cycle. Curr Top Dev Biol 49: 267–289 11005023 10.1016/s0070-2153(99)49013-1

[bib34] Stearns T (2001) Centrosome duplication. A centriolar pas de deux. Cell 105: 417–420 11371338 10.1016/s0092-8674(01)00366-x

[bib35] Stearns T, Evans L, Kirschner M (1991) Gamma-tubulin is a highly conserved component of the centrosome. Cell 65: 825–836 1840506 10.1016/0092-8674(91)90390-k

